# Weighted Lottery to Equitably Allocate Scarce Supply of COVID-19 Monoclonal Antibody

**DOI:** 10.1001/jamahealthforum.2023.2774

**Published:** 2023-09-01

**Authors:** Erin K. McCreary, Utibe R. Essien, Chung-Chou H. Chang, Rachel A. Butler, Parag Pathak, Tayfun Sönmez, M. Utku Ünver, Ashley Steiner, Maddie Chrisman, Derek C. Angus, Douglas B. White

**Affiliations:** 1 Division of Infectious Diseases, Department of Medicine, University of Pittsburgh School of Medicine, Pittsburgh, Pennsylvania; 2 Division of General Internal Medicine and Health Services Research, David Geffen School of Medicine at the University of California, Los Angeles; 3 Center for the Study of Healthcare Innovation, Implementation, and Policy, Greater Los Angeles VA, Los Angeles, California; 4 Division of General Internal Medicine, Department of Medicine, University of Pittsburgh School of Medicine, Pittsburgh, Pennsylvania; 5 Department of Biostatistics, University of Pittsburgh School of Public Health, Pittsburgh, Pennsylvania; 6 Program on Ethics and Decision Making in Critical Illness, CRISMA Center, Department of Critical Care Medicine, University of Pittsburgh School of Medicine, Pittsburgh, Pennsylvania; 7 Department of Economics, Massachusetts Institute of Technology, Cambridge; 8 Department of Economics, Boston College, Chestnut Hill, Massachusetts; 9 Department of Emergency Medicine, University of Pittsburgh School of Medicine, Pittsburgh, Pennsylvania; 10 Wolff Center for Quality and Safety, University of Pittsburgh Medical Center, Pittsburgh, Pennsylvania; 11 Department of Critical Care Medicine, University of Pittsburgh School of Medicine, Pittsburgh, Pennsylvania

## Abstract

**Importance:**

Equitable allocation of scarce medications is an important health policy goal. There are few data about attempts to achieve equitable allocation in the community setting.

**Objective:**

To describe the development and use of a weighted lottery to allocate a scarce supply of tixagevimab with cilgavimab as preexposure prophylaxis to COVID-19 for immunocompromised individuals and examine whether this promoted equitable allocation to disadvantaged populations.

**Design, Setting, and Participants:**

This quality improvement study analyzed a weighted lottery process from December 8, 2021, to February 23, 2022, that assigned twice the odds of drug allocation of 450 tixagevimab with cilgavimab doses to individuals residing in highly disadvantaged neighborhoods according to the US Area Deprivation Index (ADI) in a 35-hospital system in Pennsylvania, New York, and Maryland. In all, 10 834 individuals were eligible for the lottery. Weighted lottery results were compared with 10 000 simulated unweighted lotteries in the same cohort performed after drug allocation occurred.

**Main Outcomes:**

Proportion of individuals from disadvantaged neighborhoods and Black individuals who were allocated and received tixagevimab with cilgavimab.

**Results:**

Of the 10 834 eligible individuals, 1800 (16.6%) were from disadvantaged neighborhoods and 767 (7.1%) were Black. Mean (SD) age was 62.9 (18.8) years, and 5471 (50.5%) were women. A higher proportion of individuals from disadvantaged neighborhoods was allocated the drug in the ADI-weighted lottery compared with the unweighted lottery (29.1% vs 16.6%; *P* < .001). The proportion of Black individuals allocated the drug was greater in the weighted lottery (9.1% vs 7.1%; *P* < .001). Among the 450 individuals allocated tixagevimab with cilgavimab in the ADI-weighted lottery, similar proportions of individuals from disadvantaged neighborhoods accepted the allocation and received the drug compared with those from other neighborhoods (27.5% vs 27.9%; *P* = .93). However, Black individuals allocated the drug were less likely to receive it compared with White individuals (3 of 41 [7.3%] vs 118 of 402 [29.4%]; *P* = .003).

**Conclusions and Relevance:**

The findings of this quality improvement study suggest an ADI-weighted lottery process to allocate scarce resources is feasible in a large health system and resulted in more drug allocation to and receipt of drug by individuals who reside in disadvantaged neighborhoods. Although the ADI-weighted lottery also resulted in more drug allocation to Black individuals compared with an unweighted process, they were less likely to accept allocation and receive it compared with White individuals. Further strategies are needed to ensure that Black individuals receive scarce medications allocated.

## Key Points

**Question:**

Is development and use of a weighted lottery to promote equitable allocation of scarce resources to disadvantaged populations feasible in a large health system?

**Findings:**

In a quality improvement study examining the allocation of 450 doses of tixagevimab with cilgavimab through a weighted lottery system in a 35-instiution health system, a higher proportion of individuals from disadvantaged neighborhoods were allocated the drug in the weighted lottery compared with the unweighted lottery. Black individuals were more likely to be allocated the drug compared with an unweighted process but less likely to accept allocation and receive it compared with White individuals.

**Meaning:**

The findings of this study suggest that a weighted lottery to allocate scarce resources is feasible and may result in more drug allocation to individuals who reside in disadvantaged neighborhoods and who identify as Black; however, Black individuals allocated the drug may be less likely to accept allocation and receive it.

## Teaser

This quality improvement study examines the use of a weighted lottery system to allocate a limited number of doses of tixagevimab with cilgavimab equitably to disadvantaged populations.

## Introduction

Drug shortages occurred throughout the COVID-19 pandemic, forcing policymakers and systems to grapple with difficult decisions about who should receive treatment when not all can. Data from the US showed inequities in receipt of medications for COVID-19, with Black individuals less likely to receive treatment compared with White individuals.^1,2^ These disparities in access occurred in parallel with disproportionately higher death rates among individuals from racial and ethnic minority groups and persons with low economic resources.^3,4,5^

States promulgated a variety of guidelines for allocating scarce COVID-19 drugs.^6,7,8^ For example, the Commonwealth of Pennsylvania recommends allocating scarce COVID-19 therapeutics using a multiprinciple framework designed to promote population health outcomes and mitigate disparities across race and socioeconomic status, noting race is a social construct.^8^ A multiprinciple allocation strategy allows for incorporation of multiple ethical considerations (eg, efficacy and equity) into how a scarce resource is allocated.^9,10^ New variations of allocation schemes that accommodate multiple ethical criteria have been recently proposed, but rarely implemented.^11^ Implementing multiprinciple allocation frameworks is operationally challenging, and few empirical data exist about the use of such systems in the community setting.

Tixagevimab copackaged with cilgavimab was available under US Food and Drug Administration Emergency Use Authorization for use as COVID-19 preexposure prophylaxis in individuals aged 12 years or older with moderate/severe immune compromise from December 8, 2021, until January 26, 2023.^12^ The US government initially purchased 700 000 doses and distributed to states proportionally to case volume at no cost.

We describe the experience of a large US health care delivery system using a weighted lottery to allocate the first amount of tixagevimab with cilgavimab the system received from the government when the supply was insufficient for all eligible individuals. We describe the development of the weighted lottery process to allocate scarce tixagevimab with cilgavimab and assess whether the first round of the weighted lottery promoted equitable drug allocation to socioeconomically disadvantaged individuals and Black individuals.

## Methods

We conducted an analysis of a weighted lottery process used in the University of Pittsburgh Medical Center (UPMC) health system from December 8, 2021, to February 23, 2022. The system includes 35 hospitals, 800 outpatient facilities, and an insurance division representing 4 million individuals, predominantly located in Pennsylvania. On December 21, 2021, the Pennsylvania Department of Health notified the system it would receive 450 doses of federally allocated tixagevimab with cilgavimab. The UPMC quality review committee determined evaluation of the lottery process met quality improvement criteria and therefore was institutional review board exempt. The lottery process itself was a clinical initiative to fairly allocate a scarce medication within routine clinical care rather than a research intervention. This study follows the Standards for Quality Improvement Reporting Excellence (SQUIRE) reporting guideline.

### Development of the Allocation Process

Initial allocation would support less than 1% of eligible individuals; therefore, the system convened its clinical advisory group (CAG), comprising clinical experts, community stakeholders, and experts in community outreach, to develop an equitable allocation process that sought to accomplish 3 main ethical goals promulgated by the Pennsylvania Department of Health for allocating novel, scarce COVID-19 drugs: (1) ensure all eligible individuals have a chance to receive treatment, (2) promote community benefit, and (3) proactively mitigate health disparities in COVID-19 outcomes. The commonwealth’s guidelines do not permit direct consideration of race or ethnicity when allocating scarce COVID-19 treatments.^8^

The CAG convened rapidly by teleconference and developed the lottery process over 2 weeks. The committee considered and rejected 2 common allocation strategies: first-come, first-served and allocation based on clinician referral of individuals for treatment.^13^ The rationale was that neither approach would give all individuals a meaningful opportunity to receive treatment and would likely exacerbate, rather than mitigate, health disparities. The CAG determined the most operationally feasible strategy to achieve the commonwealth’s ethical goals was a weighted lottery among all eligible individuals within the system. Defining immunocompromise broadly, there were more than 200 000 potentially eligible individuals. The CAG then grouped immunocompromising conditions into 3 categories, with group 1 considered the most profoundly immunocompromised (eMethods in Supplement 1). Only group 1 individuals were entered into the initial lottery (n = 10 834), with the goal to extend drug allocation to every group when supply allowed.

Although the focus of this analysis is on the initial lottery, the health system received supply from the government approximately every 2 weeks and the lottery was reconducted with each supply, removing individuals who had received the drug or died and adding individuals with a new eligible diagnosis. Everyone in group 1 was eventually offered tixagevimab with cilgavimab within the lottery process. On February 23, 2022, supply had improved enough that the lottery was dissolved.

### Description of the Weighted Lottery Process

A weighted lottery gives each individual a baseline chance to receive the scarce resource while allowing the assignment of higher (or lower) chances to individuals according to specific ethical considerations.^14^ Table 1 summarizes the key attributes of the weighted lottery process and their relationship with the Commonwealth of Pennsylvania’s ethical allocation goals.

**Table 1.** Ethical Goals and Corresponding Design Elements of the Weighted Lottery Process

Ethical goal	Design element of weighted lottery process
Give all eligible individuals an opportunity to receive treatment	Proactively identify all eligible individuals via EHR searches, rather than requiring individuals to seek treatment Prohibit exclusion criteria based on the presence of severe comorbidities or disabilities Use a lottery mechanism that includes all eligible individuals Establish multiple infusion centers across geographic regions to promote meaningful access Provide transportation to/from infusion centers for individuals without access to an automobile Arrange home injection of tixagevimab with cilgavimab for individuals who cannot leave home due to frailty or disability^a^
Promote community benefit	Limit eligibility to high-risk individuals Prohibit distribution of tixagevimab with cilgavimab outside the lottery mechanism^b^
Mitigate health disparities in COVID-19 outcomes	Give higher chances in the lottery to individuals from disadvantaged neighborhoods^c^ Provide information to individuals about tixagevimab with cilgavimab using language designed for individuals with lower health literacy Locate infusion centers in or near disadvantaged neighborhoods Provide tixagevimab with cilgavimab at no cost to individuals and provide financial counsel if a co-pay was charged for injection costs and posed a financial hardship^d^

Abbreviation: EHR, electronic health record.

^a^ Originally home infusion services were designed to be available for all geographic regions, but the home infusion agency experienced severe pandemic-related nursing shortages and therefore was only able to offer home infusion services in Allegheny county.

^b^ This attribute would also prevent disparities in access if the individuals who attempted to bypass the lottery process were disproportionately of high socioeconomic status.

^c^ Giving higher chances to individuals from disadvantaged neighborhoods would also promote community benefit due to the higher average risk of infection and subsequent transmission among individuals in these neighborhoods due to greater population density, higher reliance on public transportation, and higher incidence of multigenerational households.

^d^ There was no charge to individuals for the drug costs of tixagevimab with cilgavimab. The University of Pittsburgh Medical Center Health Plan connected individuals with financial counselors to mitigate financial hardship that may arise from co-pays for injection costs for individuals in the health plan.

Two design elements were incorporated into the lottery to promote community benefit. Eligibility for the lottery was limited to individuals designated to be at the highest risk of inability to mount an immune response to vaccination (ie, group 1 individuals); every group 1 individual was automatically entered once. The health system disallowed special exceptions for lower-risk individuals to be entered into the lottery.

To mitigate health disparities in COVID-19 outcomes, individuals from socioeconomically disadvantaged neighborhoods, defined as those with a score greater than or equal to 80 on the US Area Deprivation Index (ADI),^15^ were entered into the lottery twice. The ADI ranges from 1 (least disadvantaged) to 100 (most disadvantaged) and comprises 17 education, employment, housing-quality, and poverty measures. It has been refined, adapted, and validated to the Census Block Group neighborhood level and has informed policy by associating health outcomes with residence within a disadvantaged US neighborhood.^15,16^ An ADI score of 80 or higher was chosen by the CAG based on previous data showing the most disadvantaged neighborhoods were associated with an increased risk of 30-day rehospitalization, and patients were more likely to be insured by Medicaid, have higher rates of comorbidities, live in rural areas, and identify as Black.^16^ Individuals experiencing homelessness were assigned a value of 100. The ADI score was automatically extracted from the UPMC clinical data warehouse sources when available. If the ADI score was missing from the data warehouse, it was manually entered by a member of the central team using the Wisconsin Neighborhood Mapping Tool and the patient’s address on file for every patient.^15^

The rationale for giving heightened priority (eg, 2 entries instead of 1) to individuals from disadvantaged neighborhoods was 2-fold. First, because individuals in disadvantaged neighborhoods faced a higher risk of contracting COVID-19 due to social determinants of health (eg, crowded housing, multigenerational households, and less ability to work from home), prioritizing these neighborhoods would promote community benefit.^17^ Second, because individuals living in disadvantaged neighborhoods are more likely to be from the racial, ethnic, and socioeconomic groups who have experienced a disproportionate death rate during the pandemic,^18^ prioritizing these neighborhoods may mitigate health disparities in COVID-19 outcomes.

### Other Efforts to Mitigate Disparities

The system used 8 steps to mitigate disparities and ensure that all eligible individuals had a chance to receive tixagevimab with cilgavimab. First, the system developed an electronic health record (EHR)–based dashboard to proactively identify all eligible individuals rather than relying on clinician referral. The rationale was to avoid referral bias that may pose a barrier to equal access to all eligible individuals and may exacerbate disparities. The eMethods section in Supplement 1 describes the medical coding process used to construct the dashboard. Second, the health system rejected the use of criteria to categorically exclude certain individuals from eligibility (eg, those with severe medical comorbidities or cognitive impairment).^19^ Third, the health system implemented the lottery system described above. Fourth, the health system established 22 tixagevimab with cilgavimab infusion centers spread across its service area to promote geographic equity by minimizing travel distances for individuals. Fifth, for individuals without access to transportation, the health system collaborated with a nonprofit organization (United Way) to provide transport in eligible service areas. Sixth, for individuals in southwestern Pennsylvania who were unable to leave home due to frailty or disability, arrangements were made for home injection. Seventh, staff who contacted individuals to inform them they had been allocated to receive tixagevimab with cilgavimab provided information using language designed for individuals with lower health literacy. Eighth, the federal government provided tixagevimab with cilgavimab at no cost to individuals, and the health system provided financial assistance or counselors if a co-pay was charged for injection costs that posed a financial hardship.

### Conduct of the Weighted Lottery

A central allocation team blinded to the identities and demographic characteristics of individuals conducted the weighted lottery process. Baseline lottery chances were established by dividing the number of available treatment courses (n = 450) by the number of eligible individuals (n = 10 834), with individuals from areas with high ADI or greater disadvantage included twice. Next, an online random number generator determined which individuals were allocated the drug. Two members of the allocation team observed the lottery process to ensure fidelity to the protocol.

### Notification of Individuals Who Were Allocated Tixagevimab With Cilgavimab

Trained staff members used the contact information in the EHR to contact individuals who were allocated tixagevimab with cilgavimab via telephone and used a plain-language explanation (eFigure 1 in Supplement 1). Staff members were also provided suggested answers to a list of frequently asked questions to address individual concerns (eFigure 2 in Supplement 1). The staff member determined the individual’s preferred location to receive the injection. If an individual wanted to speak with their primary care clinician before accepting treatment, drug allocation was withheld for 1 week. When an individual accepted drug allocation, the staff at the preferred infusion center subsequently contacted the individual to schedule an appointment. The central allocation team coordinated with the infusion centers and documented reasons for patient refusal of allocation via a protected health information–secured channel.

### Data Collection

The demographic characteristics (including self-reported race and ethnicity; categories may vary across differing EHRs) of all eligible individuals were obtained from the EHR. The individuals allocated tixagevimab with cilgavimab and reasons why individuals did not accept allocations were recorded in administrative records. The UPMC Clinical Data Warehouse reported which individuals ultimately received tixagevimab with cilgavimab.

### Statistical Analysis

The Fisher exact test was used to compare the proportion of Black and White individuals who resided in high ADI neighborhoods. Other races and ethnicities were not reported with enough frequency to evaluate. Individuals’ chances of drug allocation in an unweighted lottery were determined by dividing the number of available treatment courses by the number of eligible individuals. To determine whether the ADI-weighted lottery resulted in different allocation chances for individuals compared with an unweighted lottery between different groups (Black individuals compared with White individuals, or residents in high vs nonhigh ADI neighborhoods), we used a difference-in-differences method. This approach allowed us to compare each individual’s actual weighted chances with the chances they would have had in an unweighted lottery.^20^ To simulate allocation of tixagevimab with cilgavimab in an unweighted lottery, 450 individuals were randomly identified using the binomial chance of unweighted probability (450 of 10 834). Monte Carlo simulations were repeated 10 000 times and individual characteristics were averaged across the simulations. The Fisher method was used to combine *P* values for comparing individuals allocated to receive tixagevimab with cilgavimab in the weighted lottery with those in the simulated unweighted lottery. Descriptive statistics were used to summarize the reasons individuals allocated to receive tixagevimab with cilgavimab did not ultimately receive it. The Fisher exact test or the 2-sample *t* test was used to compare the demographic characteristics of individuals in different groups. Analyses were performed with Stata, version 17 (StataCorp LLC) with a significance threshold of *P* < .05 in 2-sided tests.

## Results

There were 10 834 eligible individuals, defined as those meeting immunocompromised group 1 criteria. All were entered into the initial weighted lottery for 450 courses of tixagevimab with cilgavimab. As summarized in Table 2, the mean (SD) age of eligible individuals was 62.9 (18.8) years; 5471 were female (50.5%), 5363 were men (49.5%); 767 were Black (7.1%) and 9822 were White (90.7%). Overall, 1800 eligible individuals (16.6%) lived in a disadvantaged neighborhood. Significantly more Black individuals compared with White individuals lived in a disadvantaged neighborhood (342 of 767 [44.6%] vs 1421 of 9822 [14.5%]; *P* < .001).

**Table 2.** Comparison of Demographic Characteristics of the Overall Lottery Cohort With Individuals Allocated Tixagevimab With Cilgavimab in the Weighted Lottery and a Simulated Unweighted Lottery

Variable	Lottery population (N = 10 834)	Allocated tixagevimab with cilgavimab			Received tixagevimab with cilgavimab (n = 125)
		Weighted lottery (n = 450)	Simulated unweighted lottery (n = 450)^a^		
			Mean (SD)	% (SD)	
Age, mean (SD), y	62.9 (18.8)	63.2 (17.4)	62.9 (0.9)	62.9 (0.9)	64.2 (13.6)
Sex, No. (%)					
Female	5471 (50.5)	233 (51.8)	227.2 (10.5)	50.5 (2.3)	57 (45.6)
Male	5363 (49.5)	217 (48.2)	222.8 (10.5)	49.5 (2.3)	68 (54.4)
Race and ethnicity, No. (%)					
American Indian/Alaska Native/Hawaiian/Pacific Islander	17 (0.2)	1 (0.2)	0.7 (0.8)	0.2 (0.2)	0
Asian	86 (0.8)	2 (0.4)	3.6 (1.9)	0.8 (0.4)	2 (1.6)
Black	767 (7.1)	41 (9.1)	31.9 (5.3)	7.1 (1.2)	3 (2.4)
White	9822 (90.7)	402 (89.3)	407.9 (6.1)	90.7 (1.4)	118 (94.4)
Not reported/unknown	142 (1.3)	4 (0.9)	5.9 (2.4)	1.3 (0.5)	2 (1.6)
ADI ≥80, No. (%)^b^	1800 (16.6)	131 (29.1)	74.8 (7.8)	16.6 (1.7)	36 (28.8)
Insurance status, No. (%)					
Commercial	6401 (59.1)	257 (57.1)	266.0 (10.3)	59.1 (2.3)	78 (62.4)
Medicare	2054 (19.0)	81 (18.0)	85.3 (8.2)	19.0 (1.8)	16 (12.8)
Medicaid	1082 (10.0)	54 (12.0)	44.9 (6.2)	10.0 (1.4)	11 (8.8)
Missing/unknown	1297 (11.9)	58 (12.9)	120.5 (9.2)	26.8 (2.0)	20 (16)

Abbreviation: ADI, Area Deprivation Index.

^a^ For the categorical variable, the numbers presented are the averaging count (average SD) with percentage (SD) of the count of the results from 10 000 Monte Carlo simulations.

^b^ An ADI greater than or equal to 80 defined at the census block group level indicates a disadvantaged neighborhood.

Among 450 individuals allocated to receive tixagevimab with cilgavimab in the ADI-weighted lottery, 131 (29.1%) were from disadvantaged neighborhoods. This proportion was significantly higher than the proportion of individuals from disadvantaged neighborhoods allocated to receive tixagevimab with cilgavimab in the simulated unweighted lottery (16.6% [SD, 1.7%]; *P* < .001). Furthermore, a significantly higher proportion of Black individuals was allocated to receive tixagevimab with cilgavimab through the ADI-weighted lottery (41 [9.1%]) compared with the simulated unweighted lottery (mean [SD], 31.9 [5.3]; percentage of unweighted lottery population [SD], 7.1% [1.2%]; *P* < .001).

The Figure shows the treatment status of the 450 individuals who were allocated tixagevimab with cilgavimab in the weighted lottery. Overall, 27.8% (125 of 450) of individuals who were allocated tixagevimab with cilgavimab in the lottery accepted allocation and received the drug; 40.2% (181 of 450) of individuals allocated tixagevimab with cilgavimab declined to receive it; 19.1% (86 of 450) did not respond to multiple telephone calls; 10.2% (46 of 450) were subsequently determined to be ineligible at the time of allocation (eg, current or recent COVID-19 infection or vaccination, current hospitalization, age <12 years).

**Figure.**
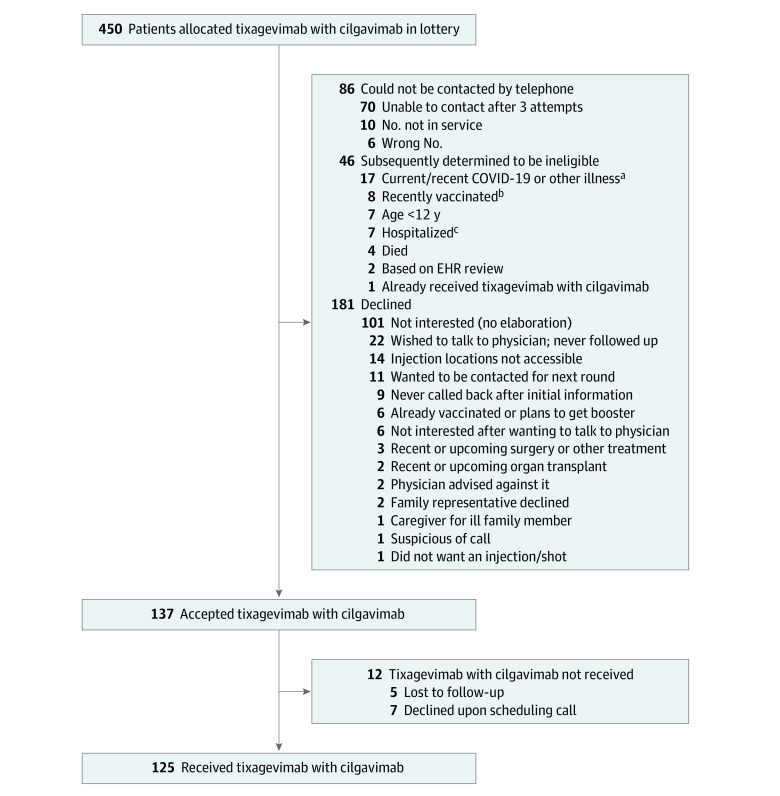
Treatment Status of First 450 Patients Allocated to Tixagevimab With Cilgavimab in Weighted Lottery EHR indicates electronic health record. ^a^At the time of the lottery, the University of Pittsburgh Medical Center health system required patients to be 20 or more days from infection and have 2 negative polymerase chain reaction tests before receiving routine outpatient treatment, due to the risk of prolonged viral shedding in this population. ^b^During the lottery period, the US Centers for Disease Control and Prevention recommended a waiting period between previous antibody therapy and/or COVID-19 vaccination and receipt of tixagevimab with cilgavimab. ^c^Patients were ineligible to receive tixagevimab with cilgavimab if they were inpatients in an acute care hospital at the time of contact.

Similar proportions of individuals from disadvantaged neighborhoods received tixagevimab with cilgavimab compared with those from other neighborhoods (27.5% [36 of 131] vs 27.9% [89 of 319]; *P* = .93) (Table 2). From the entire population, more patients from disadvantaged neighborhoods received treatment (2.0% [36 of 1800] vs 1.0% [89 of 9034]; *P* = .001). However, Black individuals who were allocated the drug were less likely to receive it compared with White individuals (7.3% [3 of 41] vs 29.4% [118 of 402]; *P* = .003). Reasons for not receiving the drug are described in Table 3; these included a lower rate of successful telephone contact of Black individuals, a lower consent rate among those contacted, and a higher rate of being ineligible for tixagevimab with cilgavimab upon contact (eg, due to having COVID-19 or being hospitalized).

**Table 3.** Receipt of Tixagevimab With Cilgavimab Among Individuals Allocated to Receive It in Weighted Lottery^a^

Variable	No. (%)			
	ADI ≥80.0 (n = 131)^b^	ADI <80 (n = 319)^b^	Black (n = 41)	White (n = 402)
Patient received tixagevimab with cilgavimab (n = 125)	36 (27.5)	89 (27.9)	3 (7.3)	118 (29.4)
Patient could not be contacted (n = 86)^c^	21 (16.0)	65 (20.4)	10 (24.4)	75 (18.7)
Patient determined to be ineligible (n = 46)^d^	18 (13.7)	28 (8.8)	7 (17.1)	38 (9.5)
Patient declined (n = 181)^e^	52 (39.7)	129 (40.4)	18 (43.9)	162 (40.3)
Tixagevimab with cilgavimab not administered (n = 12)^f^	4 (3.1)	8 (2.5)	3 (7.3)	9 (2.2)

Abbreviation: ADI, Area Deprivation Index.

^a^ No. from total population allocated (450).

^b^ An ADI level greater than or equal to 80 defined at the census block group level indicates a disadvantaged neighborhood.

^c^ Race unknown for 1 patient.

^d^ Race categorized as other for 1 patient.

^e^ Race categorized as other for 1 patient.

^f^ Tixagevimab with cilgavimab not administered: 5 individuals who previously accepted the opportunity to receive the drug did not respond to scheduling calls; 7 individuals declined upon scheduling.

## Discussion

There are 4 key findings from this study. First, a large US health care system was able to rapidly execute a systemwide regional weighted lottery for the administration of a scarce resource. Second, weighting the lottery in favor of those residing in disadvantaged neighborhoods increased their likelihood both of allocation to and receipt of the scarce resource. Third, this weighting also resulted in more drug allocation to Black individuals compared with an unweighted process. Fourth, among those allocated the drug, Black individuals were less likely to receive it.

Although not widely appreciated, scarcity of health care resources is common in the US. Awareness of scarcity increased dramatically during the pandemic, both because the demand for many COVID-19 interventions outstripped supply and because the pandemic itself compromised the supply of health care resources for other diseases and conditions. Much of the attention focused on how to end scarcity (ie, how to rapidly boost supply), but there was also considerable discussion on how best to allocate scarce resources.

The default allocation approach under scarcity is some form of first-come, first-served. This allocation approach is pragmatic in that it requires little or no infrastructure and oversight. However, there is also limited accountability, and many argue that approach is inherently unfair, exacerbating structural inequities in health care delivery, and therefore recommend allocation schemes that formally address inequity. The argument for a weighted lottery is particularly prescient in conditions, such as COVID-19, in which the incidence and likelihood of poor outcome are higher among those living in disadvantaged neighborhoods and minoritized populations.^21^ The challenge is that these alternative allocation systems require infrastructure and oversight, may be based on explicit criteria for which consensus is lacking, and may be viewed as a threat to clinicians’ obligation to promote each of their patients’ best interests.

There were several factors that likely helped UPMC deploy this weighted lottery. First, the Commonwealth of Pennsylvania had already articulated a strong vision in support of the need for allocation of scarce resources using ethical methods. Second, the health care system leadership had publicly articulated its endorsement of the equity focus espoused in the commonwealth’s vision. Third, the system had already created and empowered a systemwide committee responsible for the creation and dissemination of COVID-19 treatment guidelines, which previously gained wide cooperation from clinicians and prepared for the potential deployment of weighted lotteries of other agents earlier in the pandemic.^22^ Fourth, the system had a robust data and information technology infrastructure allowing identification of individuals and assignment of their relevant clinical and sociodemographic characteristics. Finally, the system deployed resources to support the pharmacy, clinical operations, scheduling, information technology, outreach, and communications requirements for the rollout of the lottery. Without the commitment of system leadership, resources, and prior planning, it seems unlikely the lottery would have been possible.

At the time of the lottery, tixagevimab with cilgavimab was the only drug strategy to prevent potentially life-threatening COVID-19 for immunocompromised individuals. Yet only 1 in 4 of those offered tixagevimab with cilgavimab consented to receive it. Prophylaxis is often adopted at lower rates than therapy because the benefits are less tangible. Because tixagevimab with cilgavimab was Emergency Use Authorization only and the system was contacting individuals directly, rather than via their clinicians, skepticism and unfamiliarity regarding the advantages likely also contributed to refusals. The system engaged in substantial education efforts for both individuals and clinicians and allowed patients up to 1 week to talk to their clinician before accepting or denying the allocation before the dose was reallocated. It was not feasible to deploy the lottery directly through clinicians’ offices, in part because there would be limited ability to ensure prescribing was limited to those selected by the lottery and because the primary clinician for a patient’s immunocompromising condition is not extractable from the EHR on a systemwide scale. Clinicians may also have incurred moral distress denying access to eligible patients not identified by the lottery or having to choose between patients. Although tixagevimab with cilgavimab was provided free of charge, there were charges for administration of the injection. Depending on the insurer, some of these costs would be borne by the patient, and financial concerns were cited frequently by those refusing treatment. Strategies to address each of these issues will boost the success of future lotteries.

This ADI-weighted lottery achieved statistically significant higher rates of drug allocation to Black individuals and is an example of using an indirect strategy to improve equitable access to treatment, or pharmacoequity. However, an ADI-weighted lottery will not necessarily achieve substantially more drug allocation to certain populations unless the prevalence of minoritized individuals living in these neighborhoods is markedly higher than in other neighborhoods.^23^ Black individuals allocated the drug were less likely to accept treatment, despite the health care system’s experience with and attention to community outreach to minoritized populations. This may reflect known barriers to health care access, such as digital inequity, and lower preventive care contact between clinicians and minoritized populations.^24,25^ Indeed, prior work has reported that Black patients are more likely to have incomplete medical histories in the EHR, which may have been a reason for limited contact with Black patients.^26,27^ Other studies have observed that Black and socioeconomically disadvantaged individuals may miss the potential benefits of preventive care strategies, resulting in increased mortality.^25^

### Limitations

There are limitations to this study. The lottery was repeated over several weeks, but we chose to examine only the first assignment. The interpretation of later rounds is problematic because eventually all individuals were offered tixagevimab with cilgavimab. By focusing on the first draw, we can specifically evaluate whether the intent of the lottery was met. This lottery focused on a prophylaxis strategy in a narrow patient population; it is possible that adoption rates would differ in lotteries of treatments for conditions that affect a wider proportion of the population. Ethnicity was underreported in the HER, and the association of the lottery with underrepresented racial and ethnic groups other than Black was not evaluated. Finally, the study is only a case study of 1 health care system in 1 region, and the generalizability to other health care systems and regions is unclear. Nevertheless, several of the findings will likely be of value for other systems considering lotteries in the future.

## Conclusions

In this quality improvement study, we noted that an ADI-weighted lottery process to allocate scarce resources is feasible in a large health system and resulted in more drug allocation and receipt of drug among individuals who reside in disadvantaged neighborhoods. Although the ADI-weighted lottery also resulted in more drug allocation to Black individuals compared with an unweighted process, they were less likely to receive it compared with White individuals. More effective strategies are needed to ensure that Black individuals receive scarce medications allocated. Health systems need to continue to build infrastructure wherein the treatment is brought to those who cannot access care (ie, eliminate the pharmacy desert) and continue national efforts to mitigate patient financial responsibility via federal funding programs.^28^ Equity interventions should be geared toward ensuring that disadvantaged groups have the needed knowledge of and trust in the benefits of treatment and actual opportunity to receive treatment (eg, free from serious financial and logistical barriers). Which individuals had robust access to information about tixagevimab with cilgavimab and/or a clinician and were therefore ready to make the decision at the time of allocation likely differed across sociodemographic factors. Ongoing and enhanced collaboration with community leaders and trusted representatives of minoritized communities is essential so individuals have a safe space to discuss this care and have an opportunity to have all of their concerns addressed.

## Notes

Supplement 1. **eMethods.** Clinical Categories for Lottery Prioritization **eFigure 1.** Plain-Language Explanation/Scripting for Tixagevimab/Cilgavimab Allocation (Staff Calling Patient) **eFigure 2.** Plain-Language Tixagevimab/Cilgavimab Frequently Asked Questions

Supplement 2.
Data Sharing Statement
