# Supplementary material for: Weighted Lottery to Equitably Allocate Scarce Supply of COVID-19 Monoclonal Antibody
Source: JAMA Health Forum. 2023 Sep 1;4(9):e232774. doi: 10.1001/jamahealthforum.2023.2774 (PMC10474557; doi:10.1001/jamahealthforum.2023.2774)
Supplement: Supplement 1. — eMethods. Clinical Categories for Lottery Prioritization eFigure 1. Plain-Language Explanation/Scripting for Tixagevimab/Cilgavimab Allocation (Staff Calling Patient) eFigure 2. Plain-Language Tixagevimab/Cilgavimab Frequently Asked Questions [file jamahealthforum-e232774-s001.pdf]

Supplementary Online Content

McCreary EK, Essien UR, Chang CCH, et al. Weighted lottery to equitably allocate scarce supply of COVID-19 monoclonal antibody. *JAMA Health Forum*. 2023;4(9):e232774. doi:10.1001/jamahealthforum.2023.2774

eMethods. Clinical Categories for Lottery Prioritization

eFigure 1. Plain-Language Explanation/Scripting for Tixagevimab/Cilgavimab Allocation (Staff Calling Patient)

eFigure 2. Plain-Language Tixagevimab/Cilgavimab Frequently Asked Questions

This supplementary material has been provided by the authors to give readers additional information about their work.

© 2023 McCreary EK et al. *JAMA Health Forum*.

eMethods: Clinical Categories for Lottery Prioritization

A clinical team developed a comprehensive list of immunocompromising diseases and/or medications, then divided the list into three groups, with Group 1 consisting of the most profoundly immunocompromised (i.e., most unlikely to have adequate vaccine response). Group 2 was anticipated to have variable vaccine response, and Group 3 adequate vaccine response. To promote clinical benefit, individuals from the Group 1 list were eligible for the lottery. Internal and external data published on immunophenotyping, vaccine efficacy data, and other factors were considered when determining Group status.

Group 1

- Anti-CD20/CD52/B-cell depleting therapy (e.g., rituximab, ocrelizumab, ofatumumab, alemtuzamab, belimumab)
- Bruton Tyrosine Kinase Inhibitor (BTKi) therapy (e.g., ibrutinib, acalabrutinib)
- Fingolimod, siponimod, ozanimod, ponesimod therapy
- Chimeric antigen receptor (CAR-T) therapy
- Hematopoietic cell transplant (HCT) within one year of transplant
    - Allogeneic cell transplant on immunosuppression even if greater than 1 year from transplant
- Graft versus host disease (GVHD) on therapy
- Multiple myeloma on therapy
- Chronic Lymphocytic Leukemia (CLL) on therapy
- Acute Myeloid Leukemia (AML) on therapy
- Acute Lymphocytic Leukemia (ALL) on therapy
- Solid organ transplant AND within one year of transplant or rejection treatment with thymoglobulin or alemtuzumab
- Solid organ transplant AND aged 65 years or older
- Lung transplant recipient
- Large B cell lymphoma on therapy
- Follicular lymphoma on therapy
- Severe primary immunodeficiency (i.e., common variable immunodeficiency disease (CVID), agammaglobulinemia, chronic granulomatous disease (CGD), severe combined immunodeficiency (SCID), Wiskott-Aldrich, DiGeorge, Dock 8 or Stat 3 deficiency, hypogammaglobulinemia requiring intravenous immunoglobulin (IVIG) replacement)
- Acquired immunodeficiency syndrome (AIDS) with CD4 <200 or <15%

Group 2

- All other solid organ transplant patients not meeting criteria for group 1
- All other cell transplant patients not meeting criteria for group 1
- Myelodysplastic Syndrome (MDS) on therapy
- All other hematological malignancies EXCEPT Chronic Myelogenous Leukemia and Acute Myeloid Leukemia patients who are not actively receiving treatment
- Aplastic anemia
- Age 65 years of age or older and two or more impairments of activities of daily living

© 2023 McCreary EK et al. *JAMA Health Forum*.

- All other immunosuppressive conditions receiving immunotherapy who are seronegative after 3 vaccines

Group 3

- Chronic Myelogenous Leukemia not actively receiving treatment
- Acute Myeloid Leukemia not actively receiving treatment
- All solid tumor malignancies on chemotherapy
- All other immunosuppressive conditions receiving immunotherapy
- Functional or anatomic asplenia severe combined immunodeficiency
- Sickle cell anemia
- All other primary/acquired immunodeficiency states

eMethods: ICD-10 Coding for Determining Eligible Patients

To identify immunocompromised patients in the health system, a clinical analytics team generated a comprehensive list of ICD-10 diagnosis codes and medication and procedure codes for each of the three groups. A team of pharmacists and physicians reviewed the coding for appropriateness based on Group 1 criteria. Only patients with a medical encounter within the system over the last year were included in the generated list of eligible patients. Once the preliminary list of patients in Group 1 patients was created, a pharmacist and project manager from the central team manually reviewed the list for accuracy (i.e., removing patients with erroneous coding or who were deceased). This process yielded a list of 10,834 eligible group 1 patients who were entered into the lottery.

© 2023 McCreary EK et al. *JAMA Health Forum*.

eFigure 1: Plain-language explanation/scripting for Tixagevimab/cilgavimab Allocation (Staff calling Patient)

© 2023 McCreary EK et al. *JAMA Health Forum*.

UPMC
LIFE CHANGING MEDICINE

Evusheld: Patient Initial Cold Call Script to Offer the Medicine

1/12/22

For discussion with patients. Not to be shared, posted, or distributed.

Introduction

Phone Answered:
Good morning/Afternoon. My name is ______. I am calling from UPMC. May I please speak with ____ (patient name) ____. **[Must be careful to not share PHI]**

Need to leave a voice message:
Good morning/Afternoon. My name is ______ and I am calling from UPMC to share information about a medicine for the prevention of COVID-19 called Evusheld. This message is for ____ (person's name) ____. Please call us back at this number __________ between 8am and 4pm, Monday through Friday. If we are helping others and unable to answer, please leave a message with your preferred day and time so we may call you back. Thank you. [**PHI: Be careful not to refer to them as a patient when leaving a message. Do not leave information about treatment].**

The phone was answered by someone who is not the patient:
Good morning/Afternoon. My name is ______. I am calling from UPMC to share information about a medicine for the prevention of COVID-19 called Evusheld. May I please speak with ____ (person's name) ____.

If the person is not available and they ask why you are calling, please do not share information.
I need to speak with [NAME] directly. I would appreciate you sharing my call back number with them.
[If they are not available, ask for a day/time to call, and leave the call back number.]

Conversation

I am calling from UPMC about the latest medicine that has been given Emergency Use Authorization by the FDA for prevention of COVID-19, called Evusheld. This is a monoclonal antibody that was created to prevent COVID-19 infection specifically in persons who are at high risk of getting and becoming very sick from the virus, but who may not be able to be vaccinated, or who may not create enough antibodies after vaccination.

We have received an extremely small amount of the medication and are reaching out to our patients who may benefit most from it. Our records show that you may be eligible to receive this medication. Are you interested in learning more?

NO:
OK, thank you for taking the time to talk with me. We do strongly encourage this medication. If you change your mind, you can call us back.

Yes, but I can't talk right now. Can I call you back? Yes. Please call us back. We are strongly encouraging this medication, and if we do not hear back from you, we will have to re-allocate this medication to a different patient.

Yes, but I want to talk to my family/my doctor first. OK, we understand you would like to do that. Please call us back. If we do not hear back from you, we will have to re-allocate this medication to a different patient.

YES:
Great, let me know if you need a minute to get paper and pen so you can write things down while we talk.

© 2023 McCreary EK et al. *JAMA Health Forum*.

I will share important information about Evusheld, ask you a few questions about your COVID-19 and health status, and if you are interested, we can schedule an appointment today.

Let me begin by explaining what Evusheld is. Evusheld is a medication called a monoclonal antibody that is given as two shots in your arm to help prevent COVID-19. Evusheld works by blocking the virus from entering your cells. Studies show that this is the first long-acting medication available that can protect you from severe COVID-19 for up to 6 months.

This medicine is given as 2 shots, one in each of your arms, during one clinic visit. You will be monitored for 1 hour afterward for side effects.

The current FDA allows this medicine to be given to those with specific illness or medical situations which puts them at high-risk of developing severe COVID-19 illness. It is meant to prevent illness in those who have not recently been exposed to, or who have, a current COVID-19 infection.

☐ Before I continue, are you interested in receiving the treatment?

YES:
Great! Let me ask you a few questions to see if the medication is right for you.

☐ Did you recently receive a COVID-19 vaccine?
If yes: you should wait 14 days after your COVID-19 vaccine to receive Evusheld. If you have not yet received a COVID-19 vaccine, you should wait 30 days after receiving Evusheld to receive a COVID-19 vaccine.
[If they were recently vaccinated, we will allocate them drug in the next round of lottery (once they are beyond their 14-day window). Mark this on the Excel sheet.]

☐ Do you have a current COVID-19 Infection, or have had one in the past 20 days?
If yes: If you have recently had a COVID-19 infection, you can receive Evusheld 20 or more days after you were diagnosed and are fully recovered. So that we can plan for infection prevention measures at our clinic, we will ask you to have a PCR test for COVID-19 on day 20 or more after you were diagnosed, and then another PCR test 7 days later. If both are negative, you can come to the clinic wearing a mask. If you are fully recovered but one or both of the PCR tests are positive, or you are completely asymptomatic and unable to get PCR testing, we will talk with you about what additional infection prevention measures we may need to use to keep you and all safe. Discuss with your provider about the best time to receive Evusheld. [Mark on the Excel sheet that we should follow up with them in ~3 weeks. There is no additional waiting period if they received monoclonal antibodies for COVID-19 **treatment.]**

☐ Have you been exposed to someone in the last 14 days who has tested positive for COVID-19?
If yes:

- What date were you exposed?
- Can you please explain how were you exposed? Was it someone in your household who tested positive? Were you in contact with a loved one or friend outside of your home at a social event, etc.?
- We have several sites that can give you the medicine including Pittsburgh Southside, Altoona, Hamot, Harrisburg and Williamsport. What is your preferred location for treatment?

Thank you. We will let your preferred location know so they can plan for infection prevention measures at their clinic. The clinic will call you and talk with you about what additional infection prevention measures they may need to use to keep you and all safe.
[Please document date of exposure and type of exposure (household contact, at dinner with friend, etc.). Email Snyder, Graham snydergm3@upmc.edu and McCreary, Erin mccrearye3@upmc.edu the case details. They will coordinate with local infection prevention about when to bring the patient in for an appointment.]

© 2023 McCreary EK et al. *JAMA Health Forum*.

**Thank you.** We have several sites that can give you the medicine including **Pittsburgh Southside, Altoona, Hamot, Harrisburg and Williamsport. What are your top two preferred locations for treatment? (indicate preferred locations on spreadsheet)**<br>**Great, you will be called by your preferred center to schedule you and provide further instructions.**<br>To help you plan for the best day and time, you should plan to spend about 1 ½ hours in the clinic. The shot will not take very long though you will need to stay for one hour after the shot for us to monitor you.	
**FAQs**	
Are there any side effects?	Side effects are uncommon but may include:<br>• Allergic reactions<br>• Pain, bruising, swelling, or bleeding at the injection site<br>• Headache, Fatigue, Cough
My husband is getting chemo/has an illness. Will he be called? Can he come with me to get it too?	This drug is very new, and we have been provided an extremely small amount to give. Right now, to be fair, we have randomly selected a number of our patients who would greatly benefit from this medication through an ethical lottery system. We cannot give him the medication at this time, but it **is important for you to know that everyone who is eligible to receive this medication will eventually be able to get it as we receive more medication.**
What about the pill for COVID-19? Can't I just take a pill instead if I get sick?	We believe it is always better to prevent than treat. There can also be long-term complications of COVID-19 illness, even if you are able to treat it. Therefore, prevention is the best pathway.<br><br>Though Pfizer's antiviral medicine has received emergency use authorization from the FDA, at this time, we have no information on how or when it will be available to patients.
Can I still get the COVID-19 vaccine if I get this medicine?	Yes, you can still get vaccinated after receiving this medicine. You should wait a minimum of 30 days after receiving Evusheld to receive a COVID-19 vaccine.
Will I still need to wear a mask in public if I receive this medicine?	This medication is one of several important steps to stop the spread of COVID-19. Yes, masking is still an important tool to keep you and those around you safe.
Is there a cost for this medicine?	• After receiving Evusheld, you may receive a notice from your insurance company, called an Explanation of Benefits (EOB), that a charge to give this medicine has been submitted. It is not a bill, and it is not a charge for the medicine.<br>• Though there is no cost for the medicine, there is a clinic fee for administering the medicine, which includes nursing and pharmacy time, supplies, etc. At this time, for those who do not have insurance, or if insurance does not cover the fee, you will be responsible for the fee for administering the medicine.<br>• If you receive an EOB or would like more information about any copays or deductibles you may be responsible for, please contact your health insurer and use the procedure code, M0220.
Pre-Arrival Instructions	
To prepare for your visit:	

© 2023 McCreary EK et al. *JAMA Health Forum*.

Before you leave home	☐ Please eat before you leave your home. For cleanliness, food and drinks are not allowed in the treatment areas.
Transportation and companions/support persons	We ask you to take all standard precautions:<br>☐ Always wear a mask when entering the building.<br>☐ If you can, please come alone. This will help us to limit the number of people in the clinic, allowing for proper social distancing.<br>☐ If you need assistance with driving, walking, or other help to get to your appointment we will welcome **1** companion to assist you.
Parking/Arrival/Check-In	☐ Try to arrive no sooner than 10 minutes before your scheduled appointment. This too will help us to maintain social distancing in the clinic.<br>☐ Park: _______________ (name of lot or garage, i.e. in the main lot (entrance A))

© 2023 McCreary EK et al. *JAMA Health Forum*.

eFigure 2: Plain-language Tixagevimab/cilgavimab Frequently Asked Questions

Evusheld: Patient-facing Lottery Talking Points 01/05/22

For discussion with patients. Not to be shared, posted, or distributed.

UPMC
LIFE CHANGING MEDICINE

Question	Talking Points
I heard UPMC is offering a medicine that can be used to prevent COVID-19. Is that true?<br><br>Tell me about it.	• Yes, UPMC is offering Evusheld.<br>• Evusheld is a medication called a monoclonal antibody that is given as a shot in your arm to help prevent COVID-19. EvuSheld works by blocking the virus from entering your cells. Studies show that this is the first long-acting medication available that can protect you from severe COVID-19 for up to 6 months.<br>• The current FDA has given an EUA – Emergency Use Authorization – for this medicine to be given to those with specific illness or medical situations which puts them at high-risk of developing severe COVID-19 illness.<br>• It is meant to prevent illness in those who have not recently been exposed to, or who do not currently have, a COVID-19 infection.<br>• This medicine is given as 2 shots in one visit and then you will be monitored for 1 hour afterward for any side effects.<br>• **You still need to get vaccinated if you are eligible. The vaccines are the surest way to protect yourself and others around you.**
Who is qualified to receive this medicine?	• The current FDA has given an EUA – Emergency Use Authorization – for this medicine to be given to those with specific illness or medical situations which puts them at high-risk of developing severe COVID-19 illness.<br>• It is for those 12 years and older.
I have COVID-19 but was called that I was allocated Evusheld in the lottery. What should I do?	• You can receive Evusheld after a minimum of 20 days from your COVID-19 diagnosis, if you are completely recovered at that time AND have a negative COVID-19 test. Discuss with your provider about the best time to receive Evusheld.
I have COVID-19 and received monoclonal antibodies for treatment. How long do I have to wait to receive Evusheld?	• You can receive Evusheld after a minimum of 20 days from your COVID-19 diagnosis if you are completely recovered at that time AND have a negative COVID-19 test. Discuss with your provider about the best time to receive Evusheld. There is no additional waiting period from receiving monoclonal antibodies to receiving Evusheld.
How long after Evusheld can I receive a COVID-19 vaccine?	• You can receive a COVID-19 vaccine 30 days after your Evusheld injection.
I just received a COVID-19 vaccine. When can I receive Evusheld?	• You can receive Evusheld a minimum of 14 days after your COVID-19 vaccine.
How long after Evusheld can I receive an influenza or other (non-COVID-19) vaccine?	• There is no waiting period following Evusheld before receiving another vaccine.
I currently receive a medicine for my condition (e.g., rituximab, IVIG, other	• No, you do not need to reschedule. Evusheld does not interact with those medications.

© 2023 McCreary EK et al. *JAMA Health Forum*.

monoclonal antibodies). Do I need to reschedule or hold my next treatment appointment for that medicine?	
I hear this medicine can cause heart problems. Is it safe?	• Yes, Evusheld is safe. Studies compared the number of patients who experienced a heart problem within 6 months of taking the medication, with the number of patients who did not experience a heart problem within 6 months of retaking the medication. A very small number of patients who received Evusheld had a cardiac event, and it was slightly more than those not receiving the drug.<br>• It is hard to say that this is because of the drug. All patients who had a cardiac event after taking Evusheld had a history of heart disease and health issues that put them at risk for a heart problem even without the drug.<br>• What is important to keep in mind is that if you geta COVID-19 infection there is also a risk it will cause heart problems too.
Why can't my loved one receive this medicine? They have cancer/ are immunocompromised/etc.	• This drug is very new, and we have been provided an extremely small amount to give. Right now, to be fair, we have randomly selected a number of our patients who would greatly benefit from this medication through an ethical lottery system. A lottery is the fairest way to decide who gets the medicine.<br>• This is a difficult time. We cannot give them the medication at this time, but it **is important for you to know that everyone who is eligible to receive this medication will eventually be able to get it as we receive more medication.**
A lottery? Why is there a lottery?	• We have been given an extremely small amount of the medicine. An ethical lottery is the fairest way to decide who gets the medicine.<br>• We will receive additional medication from the government. We do not yet know when that will be, but eventually all who are eligible and want the medication will be able to receive it.
It sounds like you are rationing.	• What we are doing is trying to use our limited amount of medicine in the best way possible.<br>• A lottery is the fairest way to make sure everyone is treated by the same rules. We wish we had more medicine at this time.<br>• When we receive enough supply, we will provide this medicine to all those who are eligible and want it.
Can't you get more medicine from somewhere else?	• It is not possible for us to get more right now. We believe the federal government will continue to give us more medication in the coming weeks.<br>• When we receive enough supply, we will provide this medicine to all those who are eligible and want it.
Is there a cost for this medicine?	• After receiving Evusheld, the pre-exposure prevention monoclonal antibody, you may receive a notice from your insurance company, called an Explanation of Benefits (EOB), that a charge to give this medicine has been submitted. It is not a bill, and it is not a charge for the medicine.<br>• Though there is no cost for the medicine, there is a clinic fee for administering the medicine, which includes nursing and pharmacy time, supplies, etc. At this time, for those who do not have insurance, or if insurance does not cover the fee, you will be responsible for the fee for administering the medicine.<br>• If you receive an EOB or would like more information about any copays or deductibles you may be responsible for, please contact your health insurer and use the procedure code, M0220.

For providers only; do not share with patients:
Providers with questions about Evusheld or the lottery-based allocation system may contact PreExposureMAB@upmc.edu

© 2023 McCreary EK et al. *JAMA Health Forum*.
