# Supplementary material for: Weighted Lottery to Equitably Allocate Scarce Supply of COVID-19 Monoclonal Antibody
Source: JAMA Health Forum. 2023 Sep 1;4(9):e232774. doi: 10.1001/jamahealthforum.2023.2774 (PMC10474557; doi:10.1001/jamahealthforum.2023.2774)
Supplement: Supplement 2. — Data Sharing Statement [file jamahealthforum-e232774-s002.pdf]

Data Sharing Statement

McCreary. Weighted Lottery to Equitably Allocate Scarce Supply of COVID-19 Monoclonal Antibody. *JAMA Health Forum*. Published September 01, 2023.
doi:10.1001/jamahealthforum.2023.2774

Data
Data available: No
